# Tandem Mass Tag-Based Quantitative Proteomics Analysis of Gonads Reveals New Insight into Sexual Reversal Mechanism in Chinese Soft-Shelled Turtles

**DOI:** 10.3390/biology11071081

**Published:** 2022-07-20

**Authors:** Tong Zhou, Guobin Chen, Meng Chen, Yubin Wang, Guiwei Zou, Hongwei Liang

**Affiliations:** 1 Yangtze River Fisheries Research Institute, Chinese Academy of Fishery Sciences, Wuhan 430223, China; zhoutong@yfi.ac.cn (T.Z.); chenguobin@yfi.ac.cn (G.C.); chenmeng@yfi.ac.cn (M.C.); wangyubin@yfi.ac.cn (Y.W.); 2 College of Fisheries and Life, Shanghai Ocean University, Shanghai 201306, China

**Keywords:** tandem mass tag, TMT, sexual reversal, gonad differentiation, Chinese soft-shelled turtle

## Abstract

**Simple Summary:**

The Chinese soft-shelled turtle has obvious sex dimorphism, which is an important aquatic economic species in China. Exogenous hormones can cause the sexual reversal of *P. sinensis,* but the molecular mechanism remains unclear. Here, TMT-based quantitative proteomics analysis of four types of *P. sinensis* (i.e., female (F), male (M), pseudo-female (PF), pseudo-male (PM)) gonads were performed. We found that there were common pathways, such as focal adhesion, endocytosis, apoptosis, ribosome, and spliceosome, which both played crucial roles in two comparison groups, including F vs. PM and M vs. PF. Furthermore, these differentially expressed proteins were associated with various biological processes, such as embryonic development and catabolic process, which were closely related to the sexual reversal of *P. sinensis*.

**Abstract:**

Chinese soft-shelled turtles display obvious sex dimorphism. The exogenous application of hormones (estradiol and methyltestosterone) can change the direction of gonadal differentiation of *P. sinensis* to produce sex reversed individuals. However, the molecular mechanism remains unclear. In this study, TMT-based quantitative proteomics analysis of four types of *P. sinensis* (female, male, pseudo-female, and pseudo-male) gonads were compared. Quantitative analysis of 6107 labeled proteins in the four types of *P. sinensis* gonads was performed. We identified 440 downregulated and 423 upregulated proteins between pseudo-females and males, as well as 394 downregulated and 959 upregulated proteins between pseudo-males and females. In the two comparisons, the differentially expressed proteins, including K7FKG1, K7GIQ2, COL4A6, K7F2U2, and K7FF80, were enriched in some important pathways, such as focal adhesion, endocytosis, apoptosis, extracellular matrix-receptor interaction, and the regulation of actin cytoskeleton, which were upregulated in pseudo-female vs. male and downregulated in pseudo-male vs. female. In pathways such as ribosome and spliceosome, the levels of RPL28, SRSF3, SNRNP40, and HNRNPK were increased from male to pseudo-female, while they decreased from female to pseudo-male. All differentially expressed proteins after sexual reversal were divided into six clusters, according to their altered levels in the four types of *P. sinensis*, and associated with cellular processes, such as embryonic development and catabolic process, that were closely related to sexual reversal. These data will provide clues for the sexual reversal mechanism in *P. sinensis*.

## 1. Introduction

Sexual dimorphism, which has become a hot topic in aquatic animal research, is influenced by genetics and environmental stressors [1]. The difference between males and females is one of the most common phenomena in biology that occurs in behavior and morphology [2]. Some aquatic animals, such as rice field eel (*Monopterus albus*), Chinese soft-shelled turtle (*Pelodiscus sinensis*), and half-smooth tongue sole (*Cynoglossus semilaevis*), undergo sexual reversal under certain conditions, which can lead to the redifferentiation of gonads [3,4,5]. The sex reversal process involves many important components and signaling pathways, such as sex determining region Y (*SRY*), SRY-box transcription factor 9 (*SOX9*), fibroblast growth factor 9 (*FGF9*), and R-spondin 1 (RSPO1)/WNT/β-catenin signaling pathways [6,7]. These signaling pathways and components also play crucial roles in gonadal development in vertebrates [8,9,10].

In aquatic animals, some exogenous hormones can induce sex reversal [11,12]. Li et al. found that high concentrations of endogenous estrogen can increase the probability of male-to-female sex reversal in protandrous fish, such as black porgy (*Acanthopagrus schlegeli*) and Asian seabass (*Lates calcarifer*) [12]. However, the expression level of estradiol is significantly low during female-to-male natural sex change in several fishes, such as rice field eel (*M. albus*) and grouper (*Epinephelus akaara*) [13,14]. The endogenous hormones, such as estrogen and androgen, are closely related to gonadal development, which can change the direction of gender differentiation in aquatic animals.

Chinese soft-shelled turtles, an important economic aquatic animal, display an obvious sexual dimorphism. The male individuals of *P. sinensis* have larger body size, wider calipash, less body fat, and grow faster, compared to female individuals [15]. In contrast to the turtles, such as the red-eared slider turtle (*Trachemys scripta elegans*), the sex determination type of the Chinese soft-shelled turtle belongs to the genetic sex determination type (ZW and ZZ), in which sex determination is usually determined by a single gene or combination of genes on the sex chromosomes or autosomes [16,17]. Unlike birds and mammals, in which, the master sex-determining (MSD) genes are *Dmrt1* and *SRY*, respectively, the MSD gene of reptiles remains unclear [18]. The previous research found that the sex differentiation of genetic sex-determined reptiles, such as the Chinese soft-shelled turtle, is also closely related to the RSPO1/WNT/β-catenin signaling pathway [10,19].

The sex reversal of Chinese soft-shelled turtles can be induced by exogenous hormones, such as E2 or methyltestosterone (MT), before gonadal differentiation [15]. The application of exogenous hormones will break the balance between estrogen and androgen, thereby changing the direction of sex differentiation [20]. The sex differentiation of Chinese soft-shelled turtles began on the 15th day and ended on the 22nd day, when incubated at 30 °C [21]. The pseudo-female individuals (∆ZZ) with a male genotype and female phenotype can be obtained by E2 treatment during the gonadal differentiation stage. Then, they can be crossed with the mature male individuals (ZZ) to obtain all-male offspring and increase the economics of the farming industry [22]. Therefore, research on the sex reversal process of Chinese soft-shelled turtles is used as the basis for all-male breeding.

TMT-based quantitative proteomics enables the absolute quantification of specific proteins, which is a more direct method than conventional transcriptomics [23]. It is a commonly used differential proteomics technology with high sensitivity that is widely used in research on the development and differentiation of animals and plants [24,25]. Mass spectrometry (MS)-based proteomics is a constructive approach for deciphering the molecular basis of spermatogenesis at different stages [26]. Using proteomic analysis, Feng et al. found that the ubiquitin/proteasome pathway (UPP) and cyclic AMP-protein kinase A (cAMP-PKA) signaling pathways play crucial roles during oogenesis in the Chinese mitten crab (*Eriocheir sinensis*) [27]. However, a quantitative proteomic analysis of sex reversal in Chinese soft-shelled turtle has not yet been reported. Hence, in the present study, we performed TMT-based quantitative proteomics to analyze the changes of proteins after sex reversal in *P. sinensis*. The results of the present study provide a new insight into the mechanism of sexual reversal and a theoretical basis for all-male breeding in Chinese soft-shelled turtles.

## 2. Materials and Methods

### 2.1. Chinese Soft-Shelled Turtle Husbandry

The Chinese soft-shelled turtles were acquired by Anhui Xijia Agricultural Development Co., Ltd. (Anhui, China). The temperature-controlled incubator was maintained at 30 ± 0.5 °C, with 80–85% humidity, i.e., the hatching conditions of turtle eggs. Under such incubation conditions, the gonadal differentiation period of *P. sinensis* generally starts at about 15 days after fertilization and lasts for one week [15]. The hatched turtles were kept in greenhouses with a temperature of 30 ± 0.5 °C and provided with commercial feeds three times a day.

### 2.2. Acquisition of Pseudo-Female and Pseudo-Male Individuals

Mature female and male individuals were cultured in a sunny pond with a water depth of about 1 m. E2 and MT can induce sex reversal in female or male *P. sinensis* at the embryo stage before sex differentiation [28]. E2 and MT were diluted with ethanol to 10 mg/mL. A small amount of hydrochloric acid (HCl) was applied gently to the surface of soft-shelled turtle egg using a cotton swab to soften it. A micro-syringe was then used to inject 5 µL of 10 mg/mL E2 or MT into the soft-shelled turtle eggs.

After one year, among the turtles that were induced by exogenous hormone, the physiological sex and genetic sex were identified by the tail length and sex-specific markers (4085-f/r, col-f/r) [21]. The pseudo-females had a male genotype and female phenotype, while the individuals with a male phenotype and female genotype were called pseudo-males.

### 2.3. Total Protein Extraction

The gonad tissues from four types of *P. sinensis* (ie., female, male, pseudo-female, and pseudo-male) were collected for further analysis after anesthesia using Tricaine mesylate (Tricaine methanesulfonate, TMS, MS-222, Syncaine, Tricaine-S) (Sigma, St. Louis, MO, USA) treatment. Total 12 gonad tissue samples from four types of *P. sinensis* were used to extract protein for TMT-based quantitative proteomics analysis. All samples were stored at −80 °C and each group (i.e., F, M, PF, and PM) had at least three repetitions.

The gonad samples were ground separately in liquid nitrogen and lysed with PASP lysis buffer (100 mM NH_4_HCO_3_, 8 M urea, pH 8), followed by 5 min of ultrasonication on ice. The lysates were centrifuged at 12,000× *g* for 15 min at 4 °C, and the supernatants were retained as the protein extracts. The bicinchoninic acid (BCA) protein assay kit (P0012, Beyotime, Jiangsu, China) was used to measure the concentrations of all protein samples, according to the manufacturer’s instructions.

### 2.4. Protein Digestion and TMT Labeling

Immediately use 10 mM dithiothreitol (DTT) to reduce the 100 µg of extracted protein from each sample for 1 h at 56 °C, and the proteins were subsequently alkylated with 15 mM iodoacetamide (IAM) for 1 h at 30 °C in the dark. Then, the samples were completely mixed with five volumes of precooled acetone by vortexing and incubated at −20 °C for at least 16 h. We added 100 mM triethylammonium bicarbonate buffer (TEAB) to dilute the protein samples, in order to obtain a urea in which the concentration was less than 2 M trypsin (Promega, Madison, WI, USA), and 100 mM TEAB buffer were added. Next, mix and digest the sample for 4 h at 37 °C. Finally, trypsin and CaCl_2_ were added at a 1:100 trypsin-to-protein mass ratio for a second 4 h digestion at 37 °C.

The tryptic peptides were then desalted using a Strata X C18 SPE column (Phenomenex, Torrance, CA, USA) and vacuum dried. Then, add 100 μL of 0.1 M TEAB buffer to redissolve the samples. Next, add 41 μL of acetonitrile-dissolved TMT labeling reagent (Thermo Fisher Scientific, Waltham, MA, USA) and mix the samples with shaking for 2 h at 30 °C. The reaction was stopped by adding 8% ammonia. All labeled samples were mixed with an equal volume of 30 μL, desalted, and lyophilized [29].

### 2.5. Liquid Chromatography–Tandem Mass Spectrometry (LC-MS/MS) Analysis

The lyophilized peptides were dissolved in Solvent A (0.1% formic acid/2% acetonitrile (ACN)) and directly loaded onto an analytical column (25 cm × 150 µm, 1.9 µm) constructed in our laboratory. The gradient comprised an increase from 4% to 23% solution B (80% ACN, 0.1% formic acid), and the separated peptides were analyzed using an Orbitrap Exploris 480, matched with field asymmetric waveform ion mobility spectrometry (FAIMS) (Thermo, Waltham, MA, USA), with an ion source of Nanospray Flex™ (electrospray ionization (ESI)) (Thermo, Waltham, MA, USA), spray voltage of 2.1 kV, and ion transport capillary temperature of 320 °C. The data-dependent acquisition mode was adopted for mass spectrometry; the FAIMS compensation voltages were set as 45 and 65, respectively, and followed the same acquisition parameters: full scan range of *m*/*z* 350–1500 and a resolution of 60,000 (at *m*/*z* 200). The automatic gain control (AGC) target value was auto, and the maximum ion injection time was auto. The scan-round time in MS/MS was set to 1 s, and the precursors in the full scan were selected from high to low abundance and fragmented by higher-energy collisional dissociation (HCD), where the resolution was 30,000 (at *m*/*z* 200), turbo TMT precursor fit function was turned on, and AGC target value was 1 × 10^5^. The maximum ion injection time was auto, normalized collision energy was set as 36%, intensity threshold was 5.0 × 10^3^, and dynamic exclusion parameter was 45 s. The LC–MS/MS analysis was conducted by Novogene Co., Ltd. (Beijing, China), following the approach depicted by McBride et al. [30].

### 2.6. The Identification and Quantitation of Protein

The resulting spectra from each run were searched separately against the Chinese soft-shelled turtle database (https://www.uniprot.org/uniprotkb?query=Pelodiscus%20sinensis, accessed on 9 November 2021) using the search engines: Proteome Discoverer 2.4 (PD 2.4, Thermo Fisher Scientific). The searched parameters were set as follows: the mass tolerance for precursor ions was 10 ppm, and the mass tolerance for production was 0.02 Da. The identified protein must contain at least one unique peptide. The identified peptide spectrum matches (PSMs) and proteins were retained if they had a false discovery rate (FDR) of less than 1.0%. The protein quantitation results were calculated using a *t*-test analysis. A protein whose abundance was significantly different among four groups (*p* < 0.05 and |log2Fold change (FC)C| > 1.5) was considered to be a differentially abundant protein (DAP).

### 2.7. Validation of the Proteome with RT-qPCR

In order to ensure the accuracy of the proteomic data, 9 differentially expressed genes, which were enriched in the signaling pathways related to sex reversal process, were selected for RT-qPCR. The primers were designed by Primer Premier 5 (Supplementary Table S1). The HiScript^®^ III 1st Strand cDNA Synthesis Kit (Vazyme, Wuhan, China) was used to synthesize the template cDNA. The subsequent quantification of cDNA products was performed as described previously, and relative mRNA expression levels were calculated by the 2^−(∆∆Ct)^ method [15].

### 2.8. Bioinformatic Analyses

Gene Ontology (GO) and InterPro (IPR) functional analysis were conducted using the InterPro Scan program against the non-redundant protein database (including Pfam, PRINTS, ProDom, SMART, ProSite, and PANTHER) [31]. The databases of COG (Clusters of Orthologous Groups) and KEGG (Kyoto Encyclopedia of Genes and Genomes) were used to analyze protein families and pathways. DAPs were investigated using volcano map analysis and cluster heat map analysis and enrichment analysis using GO, IPR, and KEGG [32]. The STRING-db server (http://string.embl.de/, accessed on 12 January 2022) was used to predict the protein interactions.

### 2.9. Statistical Analysis

The GraphPad Prism 8.0 software (GraphPad Inc., San Diego, CA, USA) was used to analyze all the experimental data, and each group contains three repetitions. The results are presented as mean ± SD. Student’s *t*-test was performed to compare the pairwise differences between groups.

## 3. Results

### 3.1. Acquisition of Pseudo-Female and Pseudo-Male Chinese Soft-Shelled Turtles

**Figure 1:**
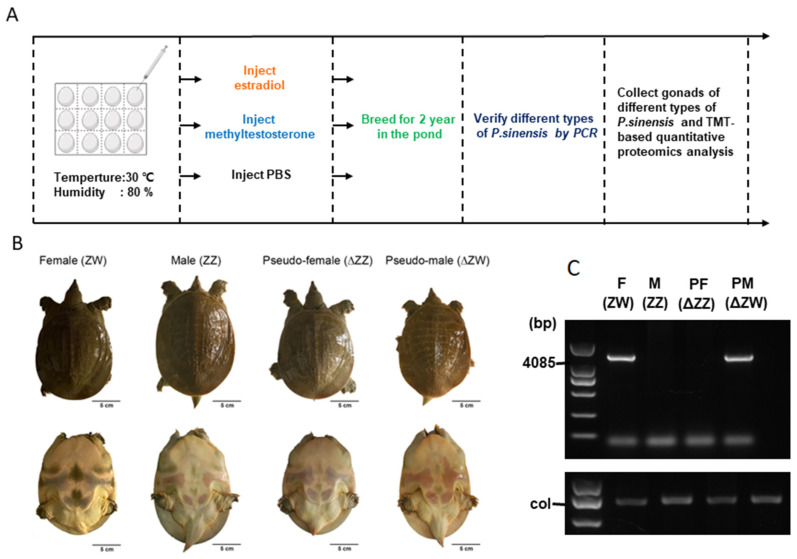
Acquisition of pseudo-female and -male of Chinese soft-shelled turtles. (**A**) The basic workflow of this experiment. (**B**) Appearances of the four types of Chinese soft-shelled turtles. (**C**) Verification of the four types of Chinese soft-shelled turtles. F: female, M: male, PF: pseudo-female, PM: pseudo-male, PBS: phosphate-buffered saline, PCR: polymerase chain reaction, TMT: tandem mass tag.

We have achieved pseudo-female and pseudo-male individuals of *P. sinensis* through a series of operations for up to 2 years (Figure 1A). The newly fertilized eggs of Chinese soft-shelled turtles cultured at 30 ± 0.5 °C began to enter the gonadal differentiation stage after 15 days. The introduction of exogenous hormones estradiol (E2) and methyltestosterone (MT) can lead to a sexual reversal in Chinese soft-shelled turtles; we collected the gonads of different types of *P. sinensis* and analyzed the changes of proteins by TMT-based quantitative proteomics. Figure 1B showed the dorsal and ventral surfaces of different types of Chinese soft-shelled turtles, and it was found that sex-reversed individuals had marked changes in tail length, relative to calipash. On the other hand, their genotypes did not change, as verified by sex-specific markers (Figure 1C, Supplementary Table S1).

### 3.2. Statistics of Quantitative Proteomics Data

The gonad tissues were collected to extract protein, enzymatically decomposed into peptide fragments, labeled with TMT, and then subjected to mass spectrometry analysis. The exported data comprised a total of 531,902 spectra generated from the gonad samples. Among them, 64,769 spectra were matched to 45,292 peptides, and 6107 proteins were identified in the gonads of *P. sinensis*.

The distribution of peptide length, protein coverage, and unique peptides showed the identification results were accurate and highly reliable (Supplementary Figure S1A,B). Supplementary Figure S1C showed that in the distribution of protein mass, and proteins > 100 kDa occupied a major part. Principal coordinates analysis (PCA) showed a clear separation of proteins in gonad tissues among the four types of *P. sinensis* (Supplementary Figure S1D). The quality control data revealed that the proteomic data met the requirements for subsequent data analysis.

### 3.3. Functional Annotation Analysis of All Samples

**Figure 2:**
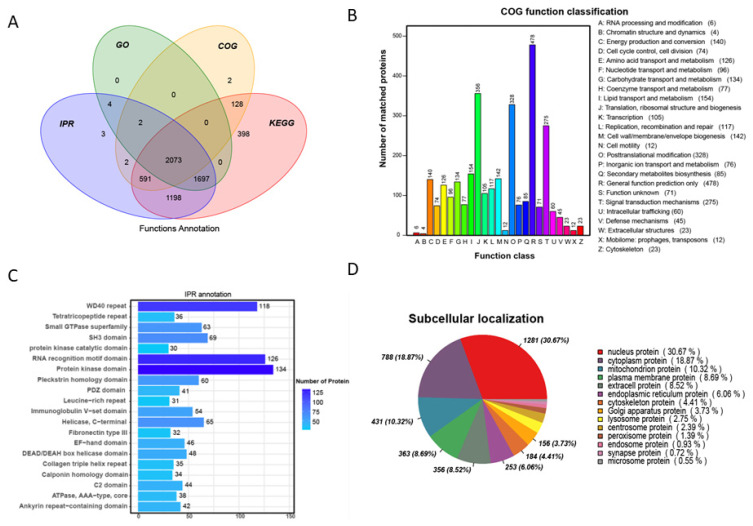
Functional annotation analysis of all samples. (**A**) Wayne analysis of annotated proteins using different databases. (**B**) COG functional classification of all matched proteins. (**C**) IPR annotation analysis of all samples. (**D**) The subcellular localization of all samples. GO gene ontology, COG: Cluster of Orthologous Groups, IPR: InterPro, KEGG: Kyoto Encyclopedia of Genes and Genomes.

All of the quantified proteins in the gonad tissues of four types of *P. sinensis* were annotated using GO, KEGG, COG, InterPro (IPR), and subcellular localization (Figure 2). The Venn diagram showed that, in the annotation of all proteins in the different databases, approximately 93.4% of the proteins were annotated by more than two databases (Figure 2A). The GO enrichment analysis of all identified proteins demonstrated that these proteins were concentrated in biological processes, such as protein and ATP binding (Supplementary Figure S2A). The proteins identified in the gonads of *P. sinensis* are mainly involved in metabolism, including lipid and carbohydrate metabolisms (Supplementary Figure S2B).

The COG database results displayed that the proteins were divided into 26 functional categories, especially in general function prediction, translation, ribosomal structure, biogenesis, and signal transduction mechanisms (Figure 2B). IPR annotation analysis mainly identified protein kinase domain, RNA recognition motif domain, and WD40 repeat-containing proteins (Figure 2C). Subcellular location analyses were performed for all identified proteins in gonads of *P. sinensis* (Figure 2D). Nuclear proteins (30.67%) and cytoplasmic proteins (18.87%) were the largest proportion among the total proteins.

### 3.4. Differentially Abundant Protein Analysis between Males and Pseudo-Females

**Figure 3:**
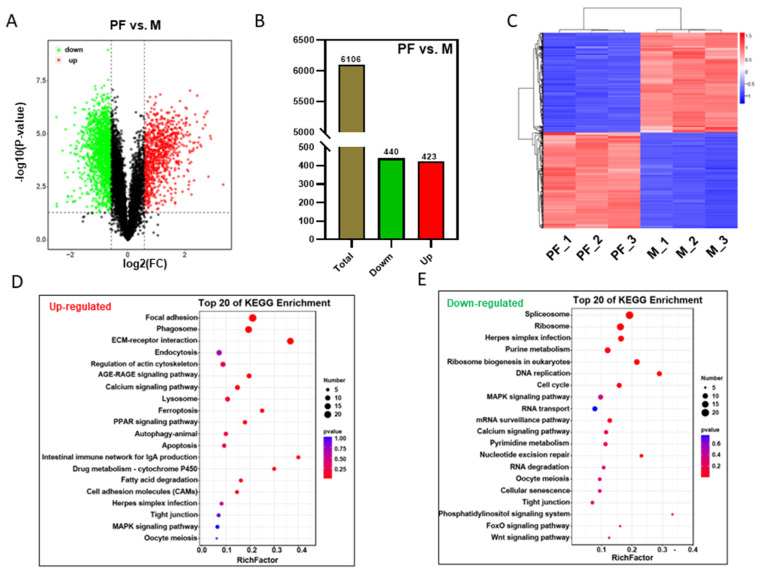
Analysis of differentially abundant proteins analysis between males and pseudo-females. (**A**) The differentially abundant proteins between pseudo-females and males. (**B**) The number of differentially abundant proteins between pseudo-females and males. (**C**) Cluster analysis of differentially abundant proteins between males and pseudo-females. (**D**) Top 20 enriched signaling pathways of upregulated proteins between pseudo-females and males. (**E**) Top 20 enriched signaling pathways of downregulated proteins between pseudo-females and males.

To analyze the changes from males (M) to pseudo-females (PF), we analyzed the differential abundant proteins. A 2.0-fold increase or decrease in the protein level and *p*-value < 0.05 were set as a threshold to identify differentially abundant proteins (DAPs). The volcano plot showed the expression level of total proteins, including up-and down-regulated proteins between male and pseudo-female (Figure 3A). The number of up-regulated proteins was 440 and down-proteins was 423 (Figure 3B). Cluster analysis of differentially expressed proteins between the male and pseudo-female showed the consistency of differentially expressed proteins among every sample, which demonstrated the reliability of the data (Figure 3C). The expression levels of the proteins, such as DYNLL1, COL4A4, ZP1, AK6, and SYF2, were significantly changed after sex reversal from male to pseudo-female. The top 30 differentially expressed proteins, including 15 upregulated and 15 downregulated proteins, were shown in Supplementary Table S2.

GO and KEGG enrichment analyses were performed on these differential abundant proteins between males and pseudo-females (Figure 3D,E, Supplementary Figure S3A). GO enrichment analysis results showed that multicellular organism development (BP), single-multicellular organism process (BP), extracellular region (CC), structural molecule activity (MF), and calcium ion binding (MF) had an effect on the sex reversal process. Among the top 20 enriched signal pathways of down-regulated proteins, focal adhesion, phagosome, and ECM-receptor interaction had the most significant changes (Figure 3D). Other enriched KEGG pathways of up-regulated proteins between M and PF covered spliceosome, ribosome, purine metabolism, and herpes simplex infection (Figure 3E). Among these differentially expressed proteins, nuclear proteins (29.04%) and extracell proteins (23.08%) were the largest proportion (Supplementary Figure S3B).

### 3.5. DAP Analysis between Females and Pseudo-Males

**Figure 4:**
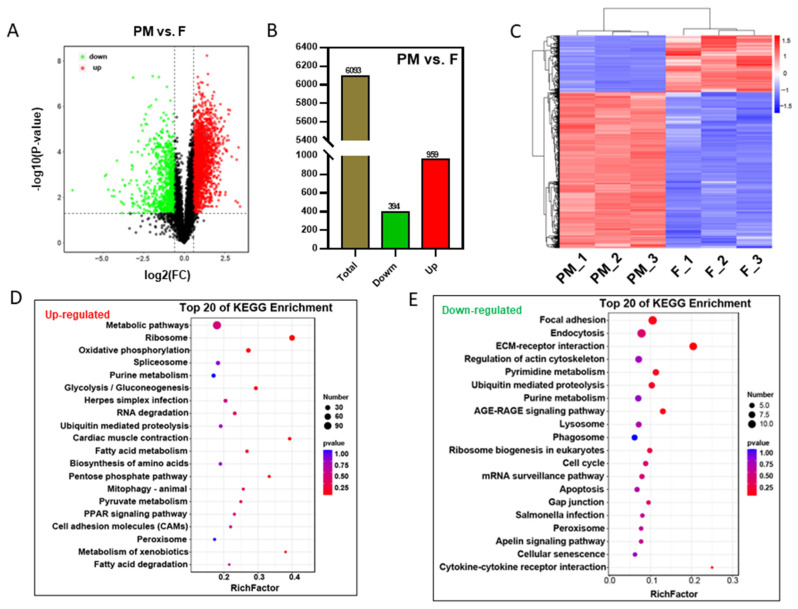
Analysis of differentially abundant proteins between females and pseudo-males. (**A**) The differentially abundant proteins between pseudo-males and females. (**B**) The number of differentially abundant proteins between pseudo-males and females. (**C**) Cluster analysis of differentially abundant proteins between pseudo-males and females. (**D**) Top 20 enriched signaling pathways of upregulated proteins between pseudo-males and females. (**E**) Top 20 enriched signaling pathways of downregulated proteins between pseudo-males and females.

We compared the DAPs between female (F) and pseudo-male (PM) individuals and constructed a volcano plot (Figure 4A). There are 394 downregulated proteins and 959 upregulated proteins among 6093 tagged proteins (Figure 4B). We performed cluster analyses for all DAPs between F and PM individuals and found that the differences among the three samples within the group were small (Figure 4C). Supplementary Table S3 showed top 30 differentially expressed proteins, such as DYNLL1, PFKP, EIF6, and BRPF3, between females (F) and pseudo-males (PM).

The biological functions and signaling pathways involving these DAPs were identified using GO and KEGG enrichment analysis (Figure 4D, Supplementary Figure S3C). Among the top 20 enriched signal pathways of the downregulated DAPs, metabolic pathways, and ribosomes have the most significantly changes. The enriched KEGG pathways of the upregulated DAPs between F and PM turtles covered focal adhesion, endocytosis, ribosome biogenesis in eukaryotes, and the cell cycle (Figure 4E). There were some differences in the subcellular location between the two groups. Among the DAPs, nuclear proteins (30.67%) and cytoplasmic proteins (20.87%) were the largest groups (Supplementary Figure S3D) between the F and PM turtles.

### 3.6. Analysis of Common KEGG Enrichment Pathways between Two Sexual Reversal Ways

**Figure 5:**
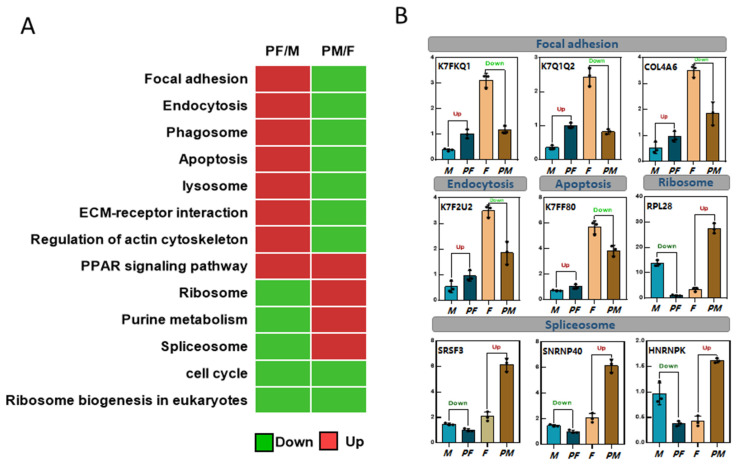
Analysis of common KEGG enrichment pathways between two sexual reversal ways. (**A**) Heatmap analysis of common KEGG pathways in two groups. (**B**) The relative expression of genes in some common pathways. Each value is presented as the mean ± SD of three repetitions. “Down” represents significantly downregulated, and “Up” represents significantly upregulated (*p* < 0.05).

By comprehensive analyses of the differentially expressed protein enrichment pathways in two groups (M vs. PF and F vs. PM), we found that some common signaling pathways were significantly changed after sexual reversal in different directions in *P. sinensis* (Figure 5). These common signaling pathways may closely related to sex reversal. Partial common signaling pathways, such as focal adhesion, endocytosis, phagosome, apoptosis, lysosome, ECM-receptor interaction, and regulation of actin cytoskeleton, showed opposite trends after sexual reversal in two groups, in which the pathways were upregulated between M and PF, but downregulated between F and PM. The DAPs associated with the peroxisome proliferator activated receptor (PPAR) signaling pathway, which were both increased in two groups. In the F vs. PM group, the DAPs that enriched in the ribosome, purine metabolism, and spliceosome pathways were significantly downregulated, while they were upregulated in the M vs. PF group (Figure 5A).

The common signaling pathways were closely related to sex reversal, and nine differentially expressed genes were selected from these pathways to detect the mRNA expression levels. We found that the change trends of some differential gene expression levels in the enriched pathways were consistent with the proteomic results (Figure 5B). The expression levels of K7FKG1, K7G1Q2, and COL4A6 (enriched in focal adhesion) were upregulated from M to PF, while they were downregulated from female to pseudo-male. K7F2U2 and K7FF80 (enriched in endocytosis and apoptosis) were significantly upregulated after sex reversal from M to PF and downregulated from F to PM. In some pathways, such as ribosome and spliceosome, the expression levels of RPL28, SRSF3, SNRNP40, and HNRNPK were all increased from M to PF, but decreased from F to PM.

### 3.7. Cluster Analysis of DAPs among the Four Types of Chinese Soft-Shelled Turtles

**Figure 6:**
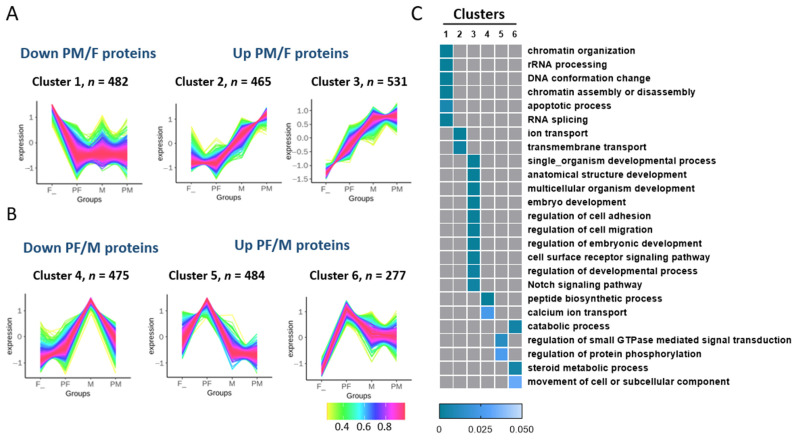
Cluster analysis of differentially abundant proteins among four types. (**A**,**B**) Fuzzy c-means clustering of regulated proteins with different kinetic profiles. Each line indicates the relative abundance of each protein. (**C**) Functional annotations of proteome clusters by GO. The GO biological process terms are displayed in heat maps, according to their statistical significance (*p* < 0.05) and locations on the GO tree.

The cluster analysis was performed on the DAPs among the different gonad tissues, which showed the changes in DAPs in every group (Supplementary Figure S4B). The proteomic results were analyzed using fuzzy c-means clustering to classify the dynamic expression levels in the gonads of Chinese soft-shelled turtles under E2 or MT. The 2714 proteins that were differentially regulated in at least one type during induction by exogenous hormones were categorized into six distinct clusters, of which, the DAPs in clusters 1 and 4 were downregulated, while those in clusters 2–3 and clusters 5–6 were upregulated in the PM vs. F or PF vs. M groups (Figure 6A,B). GO enrichment analysis showed that the proteins in the six clusters were associated with different biological processes (Figure 6C). Cluster 1 (*n* = 482) was downregulated by MT and enriched in rRNA processing, chromatin assembly and disassembly, and RNA splicing. However, the proteins in clusters 2–3 (*n* = 996; upregulated between F and PM) were specifically related to transport and development, such as transmembrane transport and embryo development. The proteins in cluster 4 (*n* = 475; downregulated in M vs. PF) were enriched in peptide biosynthetic process, calcium ion transport, and catabolic process. The proteins in cluster 5–6 (*n* = 761; upregulated by E2 in PF vs. M) were enriched in the regulation of protein phosphorylation and steroid metabolic process.

## 4. Discussion

Sexual dimorphism exists in many aquatic animals such as Japanese eel, olive flounder, and yellow catfish [33,34,35]. Chinese soft-shelled turtles, an important aquatic animal in China, show huge differences in growth and nutritional quality between male and female individuals [36]. The characteristics of male turtles are expressively better than the females, making them more popular in the market [37]. To obtain more male turtles, PF individuals were induced using E2, which can then be used to produce all-male turtles through crossing with male turtles [15]. Breeding of all male turtles has always been a hot issue in Chinese soft-shelled turtles. The gonad tissues of four type of Chinese soft-shelled turtles (i.e., M, F, PM, and PF) can develop normally and produce gametes for reproduction, as previously studied [28]. Therefore, we analyzed the changes in protein levels among four types of Chinese soft-shelled turtles (ie., M, F, PM, and PF) using TMT-based quantitative proteomics to explore the molecular mechanism of sex reversal procedure in *P. sinensis*.

In previous studies, exogenous hormones, such as estradiol and methyltestosterone, were observed to induce sex reversal in some aquatic animals [38,39,40]. Sex reversal represents an awesome sexual plasticity during the organism’s life cycle and triggers reproduction in animals [41]. Combined proteomic and transcriptomic analysis of gonadal tissue of scallops (*Chlamys nobilis*) identified 15 differentially expressed genes between the sexes, among which, 12 had evident sexual functions [42]. Chen et al. found that the proteins involved in the integrin signaling pathway, pyruvate metabolism, de novo purine biosynthesis, and ubiquitin-proteasome pathway were upregulated in the female gonads of *Acanthopagrus schlegelii* [43]. The sex reversal caused by destruction of *Kdm6b* could be rescued by *Dmrt1* in a temperature-dependent sex determination turtle species [16].

*Dmrt1* gene expression was obviously higher in males than in females in turtles such as red-eared turtles (*Trachemys scripta*) and *Lepidochelys olivacea* [44]. Liu et al. found that the expression of VASA gene is associated with sex differentiation in the Asian yellow pond turtle, *Mauremys mutica* [45]. However, Chinese soft-shelled turtles, which belong to a genetic-dependent type, were obviously different from temperature-dependent turtles, such as the red-eared slider turtle (*Trachemys scripta elegans*). In TMT-based quantitative proteomic analysis of Chinese soft-shelled turtles, we also found that there were many differentially expressed proteins that were enriched in these pathways, such as the purine metabolism and ubiquitin-proteasome pathways. The teleost fish, rice field eel, undergoes sex reversal naturally, and MS/MS analysis revealed a group of DAPs associated with ovary to ovotestis to testis transformation [46]. Meanwhile, the DAPs among different gonad tissues played different roles in sexual reversal in *P. sinensis*. In this study, we have identified 45,292 peptides and 6107 proteins in the gonads of the four types of Chinese soft-shelled turtles using TMT-based quantitative proteomics. Our results showed that both the type and number of proteins underwent dramatic changes atter sex reversal in *P. sinensis*. Therefore, the DAPs identified in gonads might play important roles in this process.

Many important proteins and pathways have crucial roles in gonad differentiation in vertebrates [12,47]. Particularly, the activation of the testicular pathways and inhibition of ovarian pathways were the primary conditions for the initiation of the male pathway in the gonads, whereas the activation of the female pathway depends on the continuous expression of the female promoter genes [48,49]. The sex reversal process of the Chinese soft-shelled turtle is mediated by E2 and MT, respectively, which determine the direction of gonadal differentiation. We found that the two sex reversal processes showed great differences and commonalities. On the one hand, there were 863 DAPs, including 440 downregulated proteins, such as eukaryotic translation initiation factor 6 (EIF6), receptor protein serine/threonine kinase (ACVR1B), and 423 upregulated proteins, such as RING-type domain-containing protein (RBX1) and calcium modulating ligand (CAMLG), which were enriched in focal adhesion, spliceosome, ribosome, apoptosis, and other pathways between F and PM. On the other hand, the 1353 DAPs, such as zona pellucida glycoprotein 1(ZP1), between M and PF were enriched in some pathways that were the same as those in F vs. PM.

The changes in key proteins in the female and male pathways might influence the direction of gonadal differentiation leading to sex reversal. The expression level of genes, such as RPL28, SRSF3, and SNRNP40, in the common pathways in Figure 5B displayed the same trends as the proteomic data. SRSF3 belongs to the serine/arginine-rich protein family, which can maintain transcriptome integrity in mouse oocytes [50]. SNRNP40 also played an important role in the male sexual differentiation and development of *M. nipponense* [51]. However, the molecular functions of these differentially expressed proteins, as related to gonad development, remain unclear, and they will be our next research direction. The common genes and pathways can deepen our understanding of the sex differentiation of Chinese soft-shelled turtles, which can guide the direction of breeding of Chinese soft-shelled turtles and increase the economics of the farming industry. The results demonstrated that some common genes and signaling pathways may both influence the sex reversal process, including from male to pseudo-female and from female to pseudo-male in *P. sinensis*.

The process of sex reversal is regulated by multiple factors [52]. Our previous studies have shown that many vital genes, such as RSPO1 and WNT4, took a dominant effect in this process [6,53]. To further understand the molecular mechanisms of sex reversal in Chinese soft-shelled turtles, we categorized the DAPs into six dynamic clusters using fuzzy c-means clustering analysis (Figure 6A), and the heat map showed the enriched GO terms for each cluster. In particular, the proteins of clusters 2–3, which were enriched in embryo development, the regulation of cell adhesion, and the regulation of embryonic development, showed significant differences between females and pseudo-males. In mice, AK1 and AK2 have been identified that they can provide a mechanism to buffer the adenylate energy charge for sperm motility in the flagellar accessory structures [54]. The aspartate kinase family proteins in cluster 4, such as adenylate kinase isoenzyme 1 (AK1, cluster 3), adenylate kinase isoenzyme 5 (AK5, cluster 3), and adenylate kinase isoenzyme 6 (AK6, cluster 6), showed significantly difference in four types of *P. sinensis*, thus playing regulatory roles in gonad development process. Taken together, the differentially expressed proteins in each cluster (1–6) were significant factors in the cultivation of the all-male Chinese soft-shelled turtles. These observations suggested that these signaling pathways might influence the gonad differentiation and provide a new insight into sex reversal in Chinese soft-shelled turtles.

## Data Availability

Data are contained within the article and Supplementary Materials.

## Supplementary Materials

The following supporting information can be downloaded at: https://www.mdpi.com/article/10.3390/biology11071081/s1. Figure S1: Statistics of the quantitative proteomics data of different samples. Figure S2: GO and KEGG enrichment analysis of labeled proteins. Figure S3: GO and KEGG enrichment analysis and subcellular location of abundant proteins in two comparison groups. Figure S4: Cluster analysis on the differentially expressed proteins among four types of Chinese soft-shelled turtles. Table S1: The primers used in this study. Table S2: Top 30 differentially expressed proteins between male and pseudo-female. Table S3. Top 30 differentially expressed proteins between female and pseudo-male.
