# Supplementary material for: Tandem Mass Tag-Based Quantitative Proteomics Analysis of Gonads Reveals New Insight into Sexual Reversal Mechanism in Chinese Soft-Shelled Turtles
Source: Biology (Basel). 2022 Jul 20;11(7):1081. doi: 10.3390/biology11071081 (PMC9312195; doi:10.3390/biology11071081)
Supplement: Supplementary file 1 [file biology-11-01081-s001.zip › biology-1724801-supplementary.pdf]

Supplementary materials

Figure S1. Statistics of the quantitative proteomics data of different samples.

(A) The distribution of peptide lengths in all samples. (B) The distribution of peptide coverage in all samples. (C) Numbers of proteins with different masses in all samples. (D) Principal coordinates analysis of four types of individuals. PC: principal coordinate.

Figure S2. GO and KEGG enrichment analysis of labeled proteins.

(A) GO enrichment analysis of labeled proteins. (B) KEGG enrichment analysis of labeled proteins.

Figure S3. GO and KEGG enrichment analysis and subcellular location of abundant proteins in two com-parison groups.

(A) GO enrichment analysis of differential proteins between male and pseudo-female. (B) The subcellular location of differential proteins between male and females. (C) GO enrichment analysis of differential proteins between female and pseudo-male. (D) The subcellular location of differential proteins between female and pseudo-male.

Figure S4. Cluster analysis on the differentially expressed proteins among four types of Chinese soft-shelled turtles.

Supplementary Table S1. The primers used in this study.

Supplementary Table S2. Top 30 differentially expressed proteins between male and pseudo-female.

Supplementary Table S3. Top 30 differentially expressed proteins between female and pseudo-male.

Figure S1

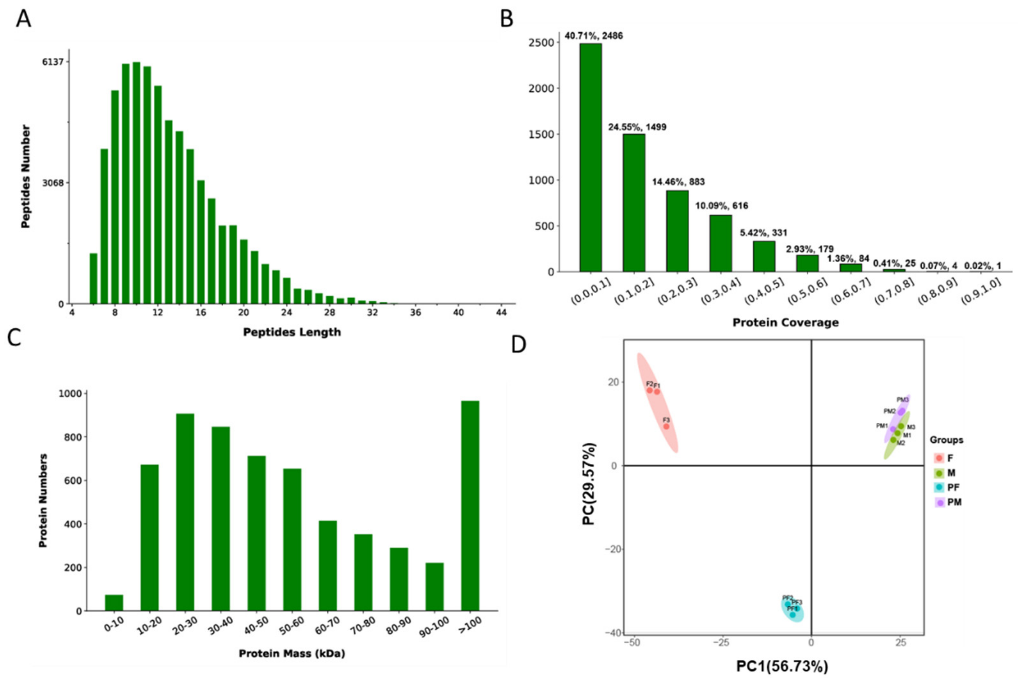
A
B
C
D
Peptides Number
Peptides Length
Protein Coverage
40.71%, 2486
24.55%, 1499
14.46%, 883
10.09%, 616
5.42%, 331
2.93%, 179
1.36%, 84
0.41%, 25
0.07%, 4
0.02%, 1
Protein Numbers
Protein Mass (kDa)
0-10
10-20
20-30
30-40
40-50
50-60
60-70
70-80
80-90
90-100
>100
PC1(56.73%)
PC(29.57%)
Groups
F
M
PF
PM

Figure S2

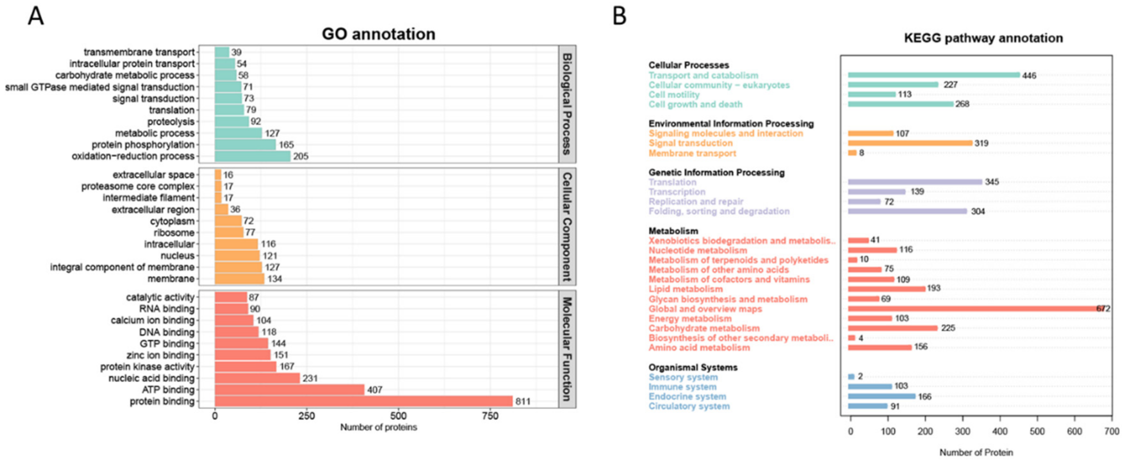
A
GO annotation
transmembrane transport
39
intracellular protein transport
54
carbohydrate metabolic process
58
small GTPase mediated signal transduction
71
signal transduction
73
translation
79
proteolysis
92
metabolic process
127
protein phosphorylation
165
oxidation−reduction process
205
Biological Process
extracellular space
16
proteasome core complex
17
intermediate filament
17
extracellular region
36
cytoplasm
72
ribosome
77
intracellular
116
nucleus
121
integral component of membrane
127
membrane
134
Cellular Component
catalytic activity
87
RNA binding
90
calcium ion binding
104
DNA binding
118
GTP binding
144
zinc ion binding
151
protein kinase activity
167
nucleic acid binding
231
ATP binding
407
protein binding
811
Molecular Function
0
250
500
750
Number of proteins
B
KEGG pathway annotation
Cellular Processes
Transport and catabolism
446
Cellular community − eukaryotes
227
Cell motility
113
Cell growth and death
268
Environmental Information Processing
Signaling molecules and interaction
107
Signal transduction
319
Membrane transport
8
Genetic Information Processing
Translation
345
Transcription
139
Replication and repair
72
Folding, sorting and degradation
304
Metabolism
Xenobiotics biodegradation and metabolis..
41
Nucleotide metabolism
116
Metabolism of terpenoids and polyketides
10
Metabolism of other amino acids
75
Metabolism of cofactors and vitamins
109
Lipid metabolism
193
Glycan biosynthesis and metabolism
69
Global and overview maps
672
Energy metabolism
103
Carbohydrate metabolism
225
Biosynthesis of other secondary metaboli..
4
Amino acid metabolism
156
Organismal Systems
Sensory system
2
Immune system
103
Endocrine system
166
Circulatory system
91
0
100
200
300
400
500
600
700
Number of Protein

Figure S3

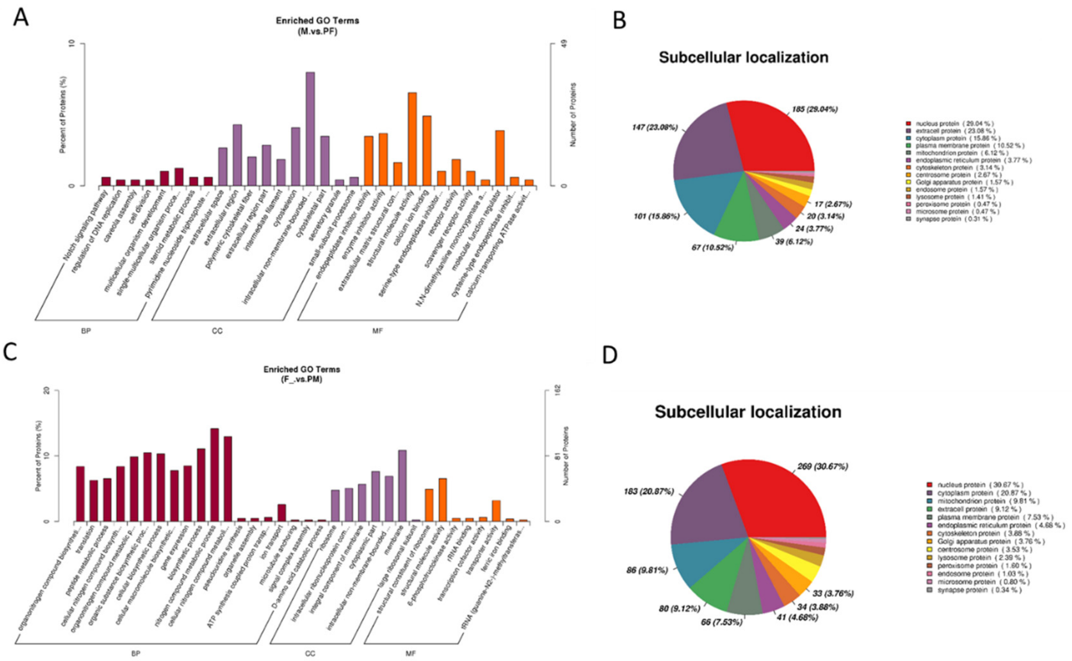
A
Enriched GO Terms
(M.vs.PF)
Percent of Proteins (%)
Number of Proteins
10
49
0
Notch signaling pathway
regulation of DNA replication
caveola assembly
cell division
multicellular organism development
single-multicellular organism proce...
steroid metabolic process
pyrimidine nucleoside triphosphate ...
extracellular space
extracellular region
polymeric cytoskeletal fiber
extracellular region part
intermediate filament
cytoskeleton
intracellular non-membrane-bounded ...
cytoskeletal part
secretory granule
small-subunit processome
endopeptidase inhibitor activity
enzyme inhibitor activity
extracellular matrix structural con...
structural molecule activity
calcium ion binding
serine-type endopeptidase inhibitor...
receptor activity
scavenger receptor activity
N,N-dimethylaniline monooxygenase a...
molecular function regulator
cysteine-type endopeptidase inhibit...
calcium-transporting ATPase activit...
BP
CC
MF
B
Subcellular localization
185 (29.04%)
147 (23.08%)
101 (15.86%)
67 (10.52%)
39 (6.12%)
24 (3.77%)
20 (3.14%)
17 (2.67%)
nucious protein (29.04 %)
extracell protein (23.08 %)
cytoplasm protein (15.86 %)
plasma membrane protein (10.52 %)
mitochondrion protein (6.12 %)
endoplasmic reticulum protein (3.77 %)
cytoskeleton protein (3.14 %)
centrosome protein (2.67 %)
Golgi apparatus protein (1.57 %)
endosome protein (1.57 %)
lysosome protein (1.41 %)
peroxisome protein (0.47 %)
microsome protein (0.47 %)
synapse protein (0.31 %)
C
Enriched GO Terms
(F_.vs.PM)
Percent of Proteins (%)
Number of Proteins
20
10
0
162
81
0
organonitrogen compound biosynthesi...
translation
peptide metabolic process
cellular nitrogen compound biosynth...
organonitrogen compound metabolic p...
organic substance biosynthetic proc...
cellular biosynthetic process
cellular macromolecule biosynthetic...
gene expression
biosynthetic process
nitrogen compound metabolic process
cellular nitrogen compound metaboli...
pseudouridine synthesis
organelle assembly
ATP synthesis coupled proton transp...
ion transport
microtubule anchoring
signal complex assembly
D-amino acid catabolic process
ribosome
intracellular ribonucleoprotein com...
integral component of membrane
cytoplasmic part
intracellular non-membrane-bounded ...
membrane
large ribosomal subunit
structural constituent of ribosome
structural molecule activity
6-phosphofructokinase activity
rRNA binding
transcription cofactor activity
transporter activity
ferric iron binding
tRNA (guanine-N2-)-methyltransferas...
BP
CC
MF
D
Subcellular localization
269 (30.67%)
183 (20.87%)
86 (9.81%)
80 (9.12%)
66 (7.53%)
41 (4.68%)
34 (3.88%)
33 (3.76%)
nucleus protein (30.67 %)
cytoplasm protein (20.87 %)
mitochondrion protein (9.81 %)
extracell protein (9.12 %)
plasma membrane protein (7.53 %)
endoplasmic reticulum protein (4.68 %)
cytoskeleton protein (3.88 %)
Golgi apparatus protein (3.76 %)
centrosome protein (3.53 %)
lysosome protein (2.39 %)
peroxisome protein (1.60 %)
endosome protein (1.03 %)
microsome protein (0.80 %)
synapse protein (0.34 %)

Figure S4

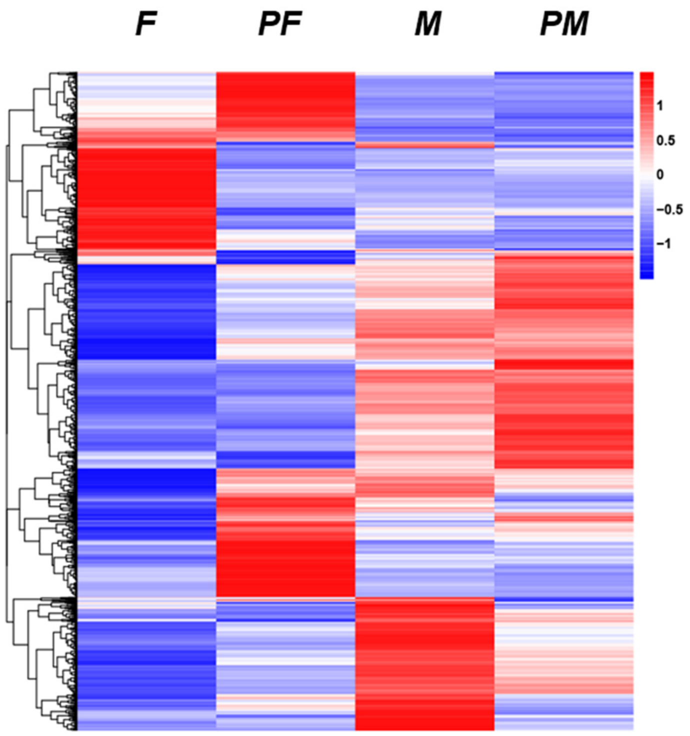

Supplement Table S1

Primers		Sequence (5′-3′)
K7FKG1	Forward	GAACAACTTCCCTGTCCTGCCTAC
	Reverse	GCCCATCTTGATGACGCTGATA
K7GIQ2	Forward	GTCCCAGAAGGCCTCCAGTGG
	Reverse	CCTCCCGTCCCAGGAACTCAC
COL4A6	Forward	CCAGCATCCAGGTGCAGATCT
	Reverse	GTGCCTTTGTAAACAGCCGAGT
K7F2U2	Forward	CTACAAATACACCCAGGAGCACC
	Reverse	AGCAGAGCCCACAGAACAGGA
K7FF80	Forward	TCATCCCGCTCCTCCTCTTTC
	Reverse	GCTCCTGTTTGCAGGCAACGT
RPL28	Forward	CGACGGCAAGGGCATTGTGGT
	Reverse	ACGCAGGCTGTTGAGGGTGGC
SRSF3	Forward	GAACAACTTCCCTGTCCTGCCTAC
	Reverse	GCCCATCTTGATGACGCTGATA
SNRNP40	Forward	GTCCTGCCTACCAACGGCTTCA
	Reverse	GCATTGCCTGTTCTTACAGACCC
HNRNPK	Forward	GGGAAAGCCCGACGGAAAGAG
	Reverse	GGTGCAGCAAAGCTATGATGACAC
4085	Forward	GTTTGAAGTGCTGCTGGGAAG
	Reverse	TTCCCCGTATAAAGCCAGGG
COI	Forward	CAACCAACCACAAAGACATTGGCAC
	Reverse	ACCTCAGGGTGTCCGAARAATCARAA

Supplement Table S2

Protein	M1	M2	M3	PF1	PF2	PF3	Up	Down
K7FE08	11.4	9.9	11.8	120.5	114	102.5	*	--
K7F5N5	93	68.3	52.4	525.5	523.5	569.8	*	--
K7FDA4	85.9	122.5	59.5	670.3	640.4	680.3	*	--
K7FJB7	89.4	83.9	94.3	673.3	607.7	624.4	*	--
TSPAN1	21.4	22.8	16	139.7	140.3	136.9	*	--
K7FTP9	96.8	113.4	79.1	630.7	648.7	643.9	*	--
ZP1	377.8	382.7	340.7	2336.1	2526.1	2393	*	--
PATL2	927.7	940.3	786.5	5466.7	5773.5	5737.2	*	--
MYL9	86.7	134.3	86.8	631.2	649	639.4	*	--
K7EW41	606.2	664.5	533.2	3640.7	3320.7	3823	*	--
RBP7	688.5	685.4	580.9	3802.3	4073.2	3558.3	*	--
LSM14B	323	287	265.9	1607.3	1765.6	1613.5	*	--
HPCAL4	763.1	729.6	658.5	3691	4075.3	4161.2	*	--
COL4A4	138.4	132.6	105.2	672.9	692.9	658.9	*	--
ACSL5	4038.8	4049.7	3492.8	18486.3	19827.2	20903.3	*	--
K7FSY5	840.5	835.3	801.3	126.1	153.5	155.5	--	*
DYNLL1	924.6	604.5	800	121.2	139.5	151.1	--	*
YBX1	1462.1	909.7	1116.7	175.5	221.6	222.5	--	*
AK6	621.1	572	663.2	107.8	137.2	133.9	--	*
K7FZ96	1308.6	1312.2	1399.2	285.9	298.2	290.4	--	*
K7G186	123.7	117.1	121	22	29.1	28.6	--	*
SARNP	648.1	490.9	706.5	121.2	146	154.2	--	*
SYF2	553.3	420.7	443.2	91.2	98.8	136.1	--	*
TCF4	188.4	173.9	146.9	38.1	40.6	40	--	*
K7EXC8	45.4	28.5	33.7	3	13.2	9	--	*
K7GE94	380.3	430.3	391.7	81	101.6	115.9	--	*
DCK	821.1	745.4	836.6	182.3	212	203	--	*
K7FRC0	880.7	720.9	911.2	209.1	216.6	214.8	--	*
ACBD7	4991.7	4541.7	6223	1333.6	1398	1433	--	*
GJC2	18.1	15.8	14.4	4.4	5.2	3.2	--	*

Supplement Table S3

Protein	F1	F2	F3	PM1	PM2	PM3	Up	Down
DYNLL1	90.5	59.1	78.1	590	1002.3	892.9	*	--
K7FX63	86.2	59.6	68.2	665.3	728.1	793	*	--
K7EXP1	42.3	46.8	55.7	365	510.2	554	*	--
K7FHJ3	98.2	59.6	82.7	665.6	763.8	845.5	*	--
K7G5Y2	38.4	36.5	50.7	322.7	353.7	461.8	*	--
PFKP	44	24.7	38.2	268.6	245.5	301.2	*	--
RBM45	89.2	83.2	77.7	570.7	607.9	589.3	*	--
LDHA	316.7	243	262.7	1751.4	1980.5	1787.9	*	--
K7EZ03	409.8	328.8	340.8	2333.3	2484.1	2409.9	*	--
RNLS	59.8	59.1	76.1	418.7	438.3	426.6	*	--
ATP6	29	21.6	40.3	197.7	186.4	201	*	--
RBX1	223.7	185.5	253.5	1234.9	1466.9	1413.5	*	--
PIH1D1	26	19.5	36.2	158.8	172.9	172.9	*	--
K7G186	18.8	14.9	27.8	115.6	123.4	133	*	--
CAMLG	42.7	25.2	48.2	214.6	234.2	248.7	*	--
DENND4A	11927.4	14986.1	12737.7	119.1	116.5	99.1	--	*
EIF6	5740	5401.6	6056.9	204.3	177.8	200.8	--	*
BRPF3	1190.5	1165.8	1287.6	44.4	41.3	44.4	--	*
ARHGAP5	766.2	876.5	701.2	33.5	26.3	31.5	--	*
POLR2H	2315.7	2168.3	1702.4	99.6	90.1	93	--	*
HDGFL2	7703.2	7421.9	6167.5	369.1	339.4	333.7	--	*
K7FDG6	566.9	688	778.5	37.9	32.5	32.8	--	*
K7GG70	967516.7	1019208	1004697	58918.2	54339.6	55180.5	--	*
K7GGJ3	134159.5	145429.5	169903.3	10049.1	9405	9237	--	*
K7GID9	10766.8	13260.2	8832.9	729	724.7	748.2	--	*
K7F1A5	922.4	800.5	416	56.4	46.6	46.5	--	*
K7GEE1	885.3	949	615.5	57.8	60.6	56.8	--	*
CEP20	1721.1	1878	1652.5	126.3	129.7	123.3	--	*
ARL5A	814.9	974.2	606	66.3	56.7	59.7	--	*
PM20D2	17025.8	16955	14568.5	1226.7	1288.2	1331	--	*
